# Comparison of wavelet transformations to enhance convolutional neural network performance in brain tumor segmentation

**DOI:** 10.1186/s12911-021-01687-4

**Published:** 2021-11-23

**Authors:** Mohamadreza Hajiabadi, Behrouz Alizadeh Savareh, Hassan Emami, Azadeh Bashiri

**Affiliations:** 1 Brain and Spinal Cord Injury Research Center, Neuroscience Institute, Tehran University of Medical Sciences, Tehran, Iran; 2 National Agency for Strategic Research in Medical Education, Tehran, Iran; 3 Shiraz University of Medical Sciences, Shiraz, Iran; 4 Faculty of Allied Medical Sciences, Shahid Beheshti University of Medical Sciences, Tehran, Iran; 5 Department of Health Information Management, School of Health Management and Information Sciences, Health Human Resources Research Center, Clinical Education Research Center, Shiraz University of Medical Sciences, Shiraz, Iran

**Keywords:** Brain, Tumor, Segmentation, MRI, Convolutional neural network

## Abstract

**Introduction and goal to background:**

Due to the importance of segmentation of MRI images in identifying brain tumors, various methods including deep learning have been introduced for automatic brain tumor segmentation. On the other hand, using a combination of methods can improve their performance. Among them is the use of wavelet transform as an auxiliary element in deep networks. The analysis of the requirements of such combinations has been addressed in this study.

**Method:**

In this developmental study, different wavelet functions were used to compress brain MRI images and finally as an auxiliary element in improving the performance of the convolutional neural network in brain tumor segmentation.

**Results:**

Based on the results of the tests performed, the Daubechies1 function was most effective in enhancing network performance in segmenting MRI images and was able to balance the performance and computational overload.

**Conclusion:**

Choosing the wavelet function to optimize the performance of a convolutional neural network should be based on the requirements of the problem, also taking into account some considerations such as computational load, processing time, and performance of the wavelet function in optimizing CNN output in the intended task.

## Introduction

Brain tumors are dangerous diseases caused by an abnormal division of cells in the brain [1–3]. Unlike most other tumors, given the value and importance of the brain in the body, both benign and malignant types are dangerous [4, 5]. Along with the methods used to diagnose the brain tumor, MRI imaging is an important still and image segmentation is one of the main processes in MRI image analysis, which is used to extracting tumor size, also visualization and display [6–12]. Accurate brain tumor segmentation is useful for brain modeling and pathological atlases generation [3, 13].

Brain MRI segmentation is a challenging task because differences in size, shape, texture, and brightness of tumors in images increase the probability of error occurrence [14]. Also large numbers of brain scans increase the time of MRI analysis [15]. The mentioned factors turn brain MRI segmentation into a complex and time-consuming process that results in wrong or delayed decisions [16, 17]. Various experiences have been presented in the papers to automate brain tumor segmentation which involves the application of a variety of numerical methods and machine-learning techniques to the processing of MRI images [4, 18–26].

Authors in [19, 27, 28] introduced a new algorithm for automatic brain segmentation based on intensity inhomogeneity and noises in brain MR images. They worked on a nonlocal means technique to reach an spatially regularized segmentation method. Increasingly used deep learning methods were also applied for tumor detection [20–24, 26, 29–32]. Authors in [33] used differential feature neural network and symmetry axes of brain MRI images via a 2D CNN to identify tumor section in MRI slices.

Also, some another deep learning methods such as convolutional neural networks have been introduced (CNN) in this area [31–36]. Unlike older methods, in which feature engineering plays a key role in the success of the method, deep learning methods don’t require feature engineering. They acquire hierarchical attributes and features directly from raw data without feature engineering [37, 38] that lead to a high-level data representation based on a hierarchy of middles [39, 40].

On the other hand, one of the most common transformations used in signal and image processing is wavelet transform. Its main capability is to parse input signal at different scales of details and approximations. So, it is known as a multispectral analysis tool, capable of extracting information from signals at different levels [41–45]. Using wavelet transforms to enhance the CNN performance requires applying some considerations such as the type of kernel function and the number of wavelet decomposition levels that are the subject of the present study. This is a continuation of our previous study [11] and involves a comparative analysis of wavelet transforms in improving CNN performance in brain tumor segmentation.

## Materials and methods

This developmental study was approved and supported by the Tehran University of Medical Sciences with the project code: 96-04-85-36667 and ethical approval No: IR.TUMS.VCR.REC.1396.4506.

In order to compare wavelet transformations, as the aim of this study, the BRATS dataset was used. It includes a total of 227 brain tumor MRI images, consisting of 227 cases of high-grade glioma and 50 cases of low-grade glioma. Details of accessing and using BRATS 2015 and some extra descriptions were explained in detail in [11], our previous study.

Multimodal Brain Tumor Image Segmentation Benchmark (Brain Tumor Segmentation: BRATS), the dataset used in this study, has been validated and aggregated by NIH radiologists in order to compare the state-of-the-art in automated brain tumor segmentation and highlight their performance. It has been organized from 2012 as BRATS challenge in conjunction with the international conference on Medical Image Computing and Computer Assisted Interventions (MICCAI). For this purpose, a unique dataset of MR scans of low- and high-grade glioma patients made available by several human experts, as well as realistically generated synthetic brain tumor datasets for which the ground truth segmentation is known. Each automatic brain tumor segmentation algorithm can use this dataset as its present its performance, exactly for dataset goal as a benchmark for comparing with others [46].

As described in [11], to compare the CNN’s performance enhancement via different wavelet transform’s in brain tumor segmentation, their output was injected into the CNN’s structure.

### Multiple wavelet injection

A Fully Convolutional Network (FCN) model in U-Net form was used for segmentation in this study. In addition to the fact, that many studies have used this method in segmentation applications, the following reasons for using FCN were compelling. FCN U-Net, due to its unique features in compressing and decompressing feature maps, is a good choice for the application of segmentation. In addition, FCN U-Net is capable to accept additional feature maps (wavelet transformations) at multiple layers.

FCN structures involve three types of layers: convolution, inverse-convolution, and pooling (sampling). The basis of this network’s function is compressing the input images into smaller feature maps till the middle layers, then reconstructing the output (segmented) image in the tail layers by up sampling and reverse-convolution of small feature maps [47].

**Figure1:**
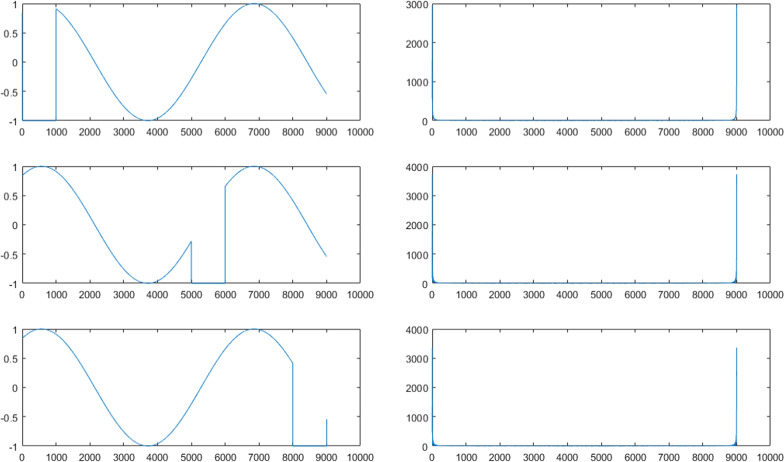
Left: original signals, Right: Fourier transform of signals

In the case of wavelet transforms, it should be noted that wavelets are mathematical transformations that break down the input signal into different frequencies with appropriate levels of details and approximation. Their advantage over Fourier transforms is their simultaneous decomposition of frequency and time [48]. Whereas Fourier transforms are not capable of extracting events location, and just provide frequency information [49]. Figure 1 shows an example case where the change of event location does not affect the Fourier transform output.

**Fig. 2:**
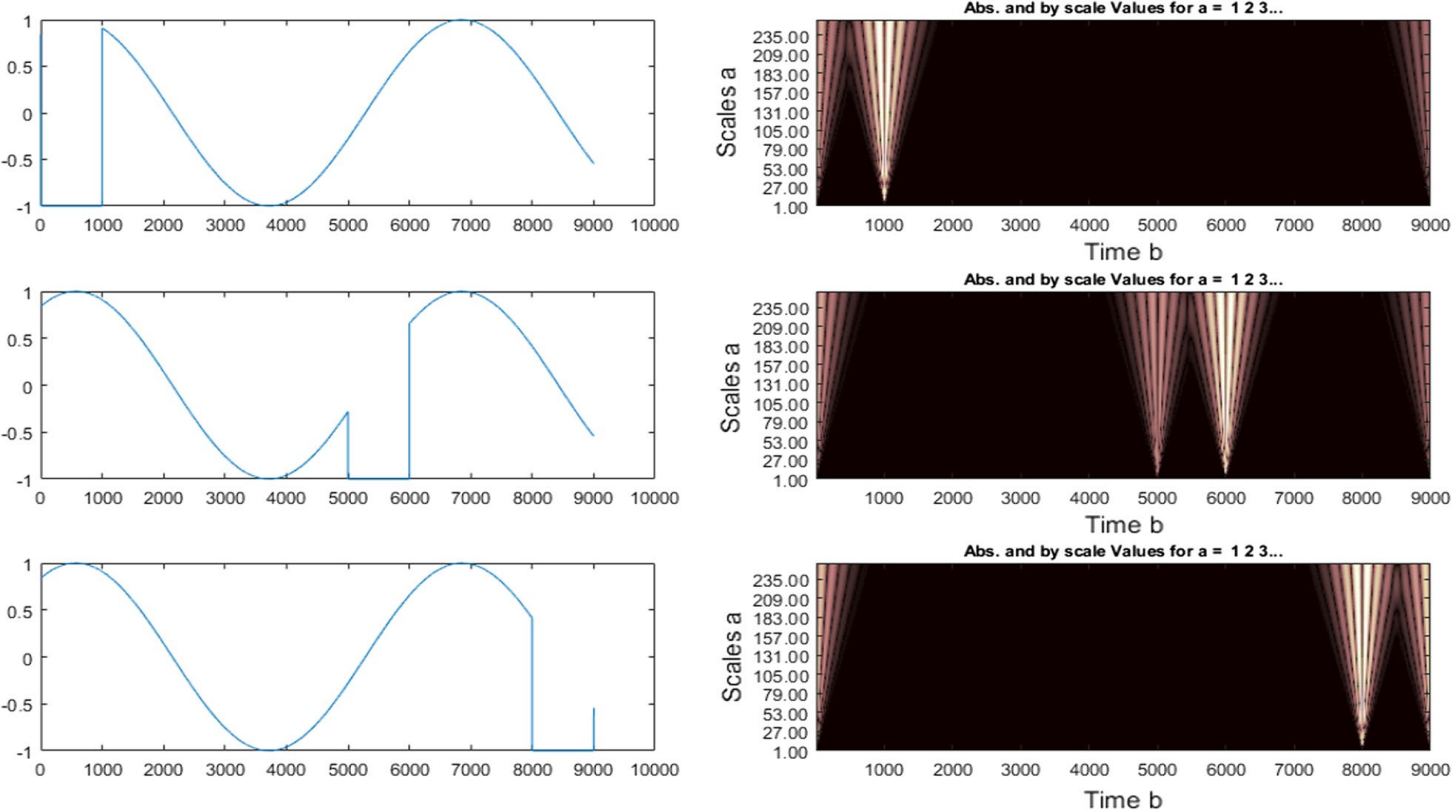
Left: original signals, Right: Wavelet transform of signals

Parsing the same signals using wavelet transform gives quite different results as Fig. 2.

**Fig. 3:**
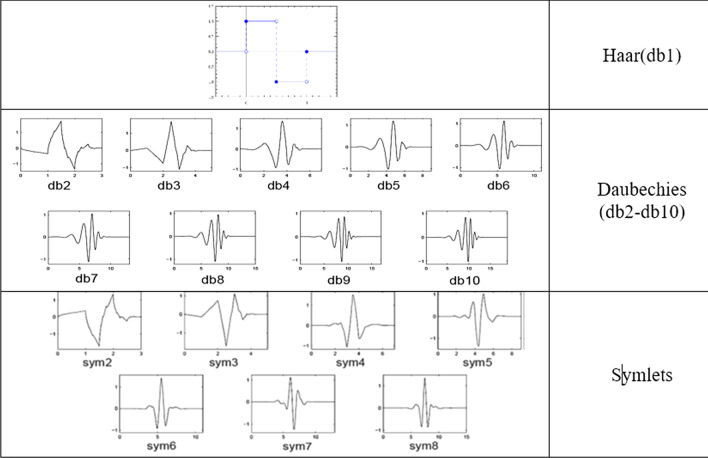
Types of wavelet transformations

Using wavelet transformations in signal analysis requires some special conditions and considerations as mentioned earlier. Based on the definitions, wavelet is a function that has several important properties: oscillation, zero mean and short length. In order to be applicable, it is necessary to concentrate on a limited range (-k, k) [50, 51]. However, the number of wavelets is infinite with this definition [52]. Some famous types of wavelets are listed in Fig. 3 [53].

In this study, a variety of wavelet transforms were used to improve CNN performance and their role in CNN segmentation performance was investigated too. During the comparison, the Pywt library was used to implement the wavelet transformation and Tensorflow library was used to implementation of CNN’s structure.

### Training WFCN network

According to the purpose of the study to identify tumor areas against other parts of the image, the two-class network mode was used in the form of binary decisions (soft_max). In order to speed up the calculations and strengthen the network performance, the default network weights adopted from imagenet-vgg-verydeep-19. In order to train the convolutional neural network, in this study, the backpropagation algorithm with a number of executions of 100 epoch was used. The use of this number of training rounds was considered according to the trial and error and due to the relative instability of the amount of errors in the epoch above 80. The training was performed on hardware equipped with Nvidia GTX 980Ti, each time it took about 18 h to run epochs.

### Performance evaluation

The evaluation of algorithms in brain tumor segmentation was performed using Dice metric which is a measure of coincidence between the segmented area and actual tumor area as below.

$${\varvec{D}}{\varvec{i}}{\varvec{c}}{\varvec{e}}=\frac{2*|{\varvec{S}}\cap {\varvec{G}}|}{|{\varvec{S}}|+|{\varvec{G}}|}$$

S is the pixels of the tumor segmented by the algorithm and G is the pixels of the actual tumor.

## Results

According to the definition of wavelet transforms, there were a plenty of options for wavelet injection such as daubechies, Morlet, Symmlet and more [54]. In order to select the appropriate wavelet, transform to compress input images, a comprehensive review performed.

**Table:** 

'db1','db2', 'db3', 'db4', 'db5', 'db6', 'db7', 'db8', 'db9', 'db10', 'db11', 'db12', 'db13', 'db14', 'db15', 'db16', 'db17', 'db18', 'db19', 'db20', 'db21', 'db22', 'db23', 'db24', 'db25', 'db26', 'db27', 'db28', 'db29','db30', 'db31', 'db32', 'db33', 'db34', 'db35', 'db36', 'db37', 'db38', 'sym2', 'sym3', 'sym4', 'sym5', 'sym6', 'sym7', 'sym8', 'sym9', 'sym10', 'sym11', 'sym12','sym13', 'sym14', 'sym15', 'sym16', 'sym17', 'sym18', 'sym19', 'sym20', 'bior1.1', 'bior1.3', 'bior1.5', 'bior2.2', 'bior2.4', 'bior2.6', 'bior2.8', 'bior3.1', 'bior3.3', 'bior3.5', 'bior3.7', 'bior3.9', 'bior4.4', 'bior5.5', 'bior6.8', 'rbio1.1', 'rbio1.3', 'rbio1.5', 'rbio2.2', 'rbio2.4', 'rbio2.6', 'rbio2.8', 'rbio3.1', 'rbio3.3', 'rbio3.5', 'rbio3.7', 'rbio3.9', 'rbio4.4', 'rbio5.5', 'rbio6.8', 'dmey'	

The selection of the wavelet function should be done in a way that is suitable in tumor identification. As one of the main features of tumors in MRI images is their brightness difference to the background, therefore, edge detection is a useful clue for tumor segmentation. Also, given the high process requirements of deep learning methods, it was necessary to select a function as the wavelet kernel that is simple as possible in terms of computational burden. Table 1 shows some examples of image compression using different wavelet transforms. The table contains examples of the application of various types of wavelet transformations in the input image compression. As in the table show, there is an image in each cell that shows how to compress MRI images. Each image is made of a structure of 4 columns * 5 rows. The columns are in T1, T2, T1 with contrast (T1C), Flair order and, from left to right. The last row of the image is ground truth shows the location of the tumor.

**Table.1:**
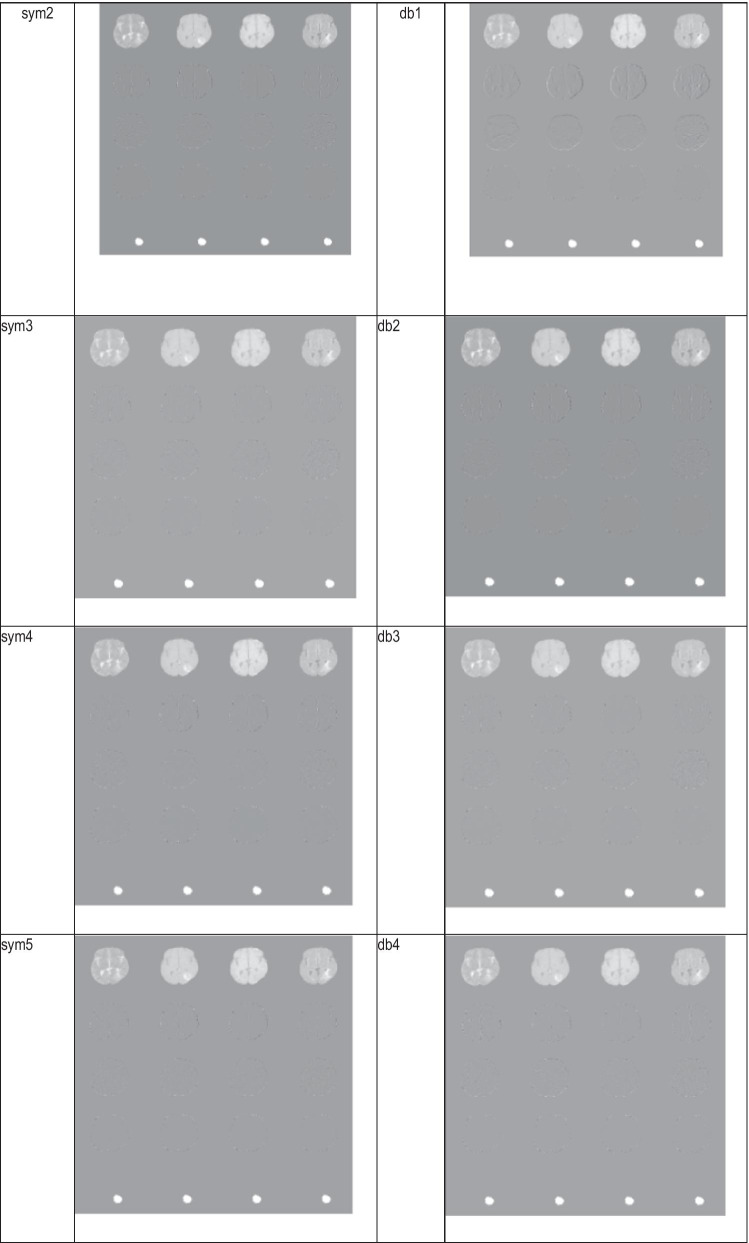
Compression of tumor images by wavelet transform Left to right: Flair, T1C, T2, T1 Top to bottom: image approximation, horizontal, vertical and diagonal details

**Fig. 4:**
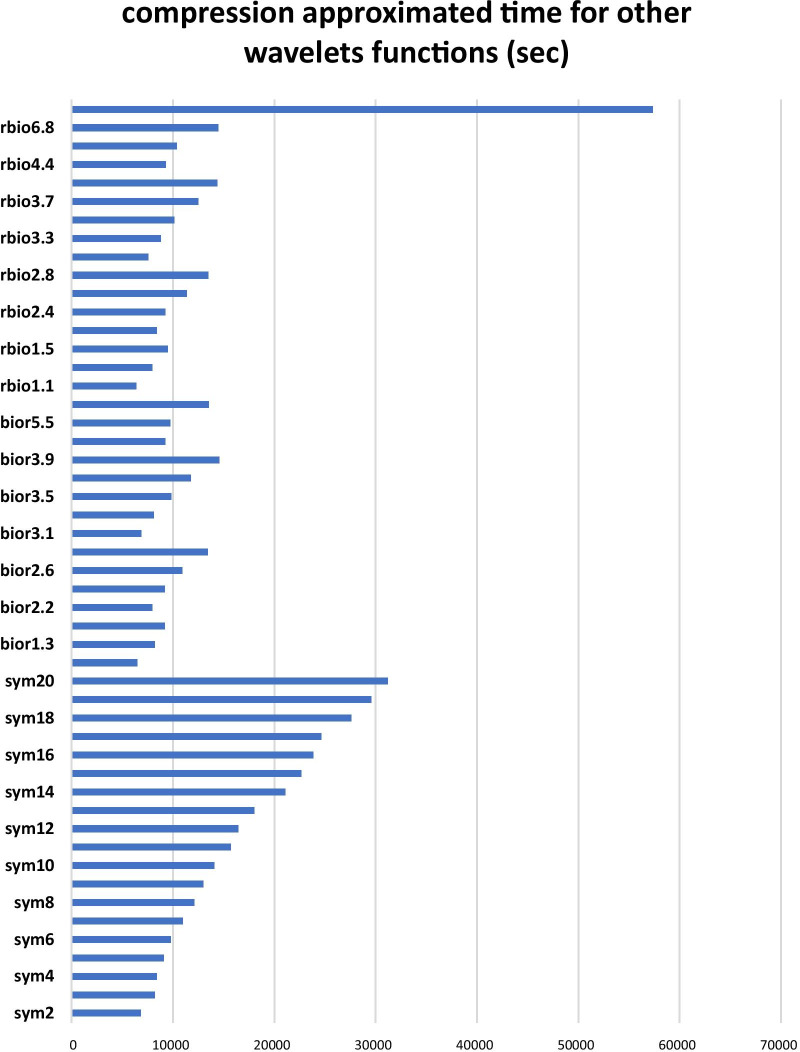
Comparative diagram of compression time of brain tumor images for non-daubechies wavelet transforms

**Fig. 5:**
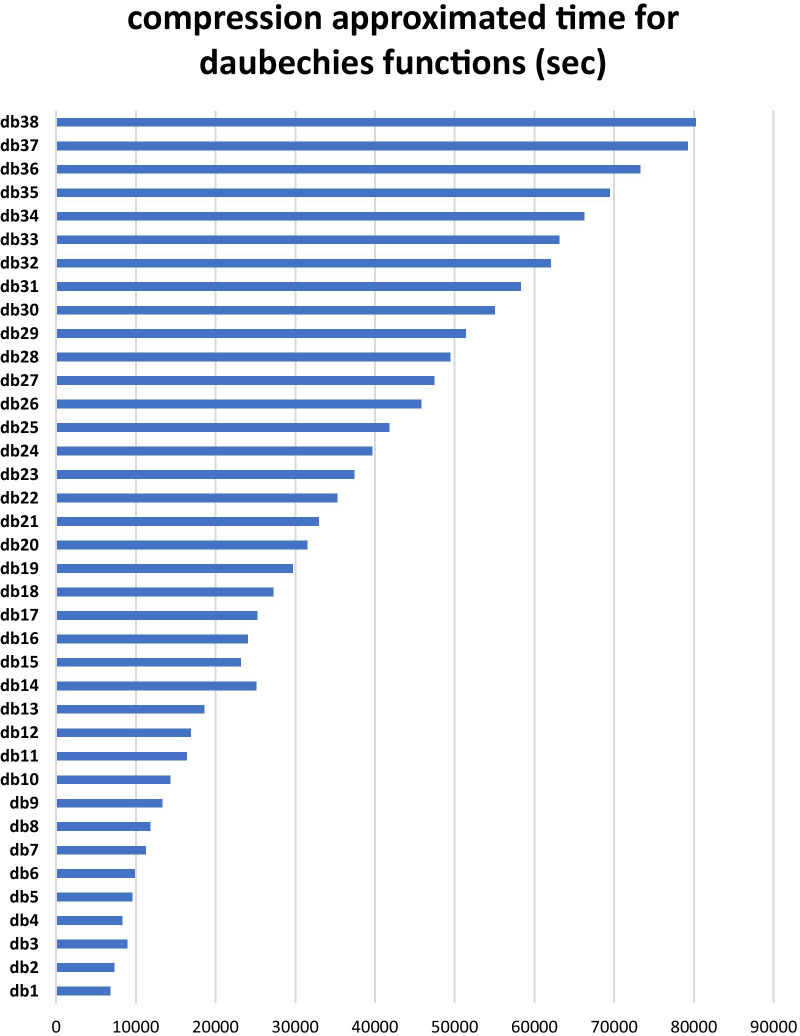
Comparative diagram of time of compression for brain tumor images using daubechies wavelet transforms

Also the average time consumed for compressing input images are presented in the Fig. 4 (non daubechies kernels) and Fig. 5 (for daubechies kernels).

**Fig. 6:**
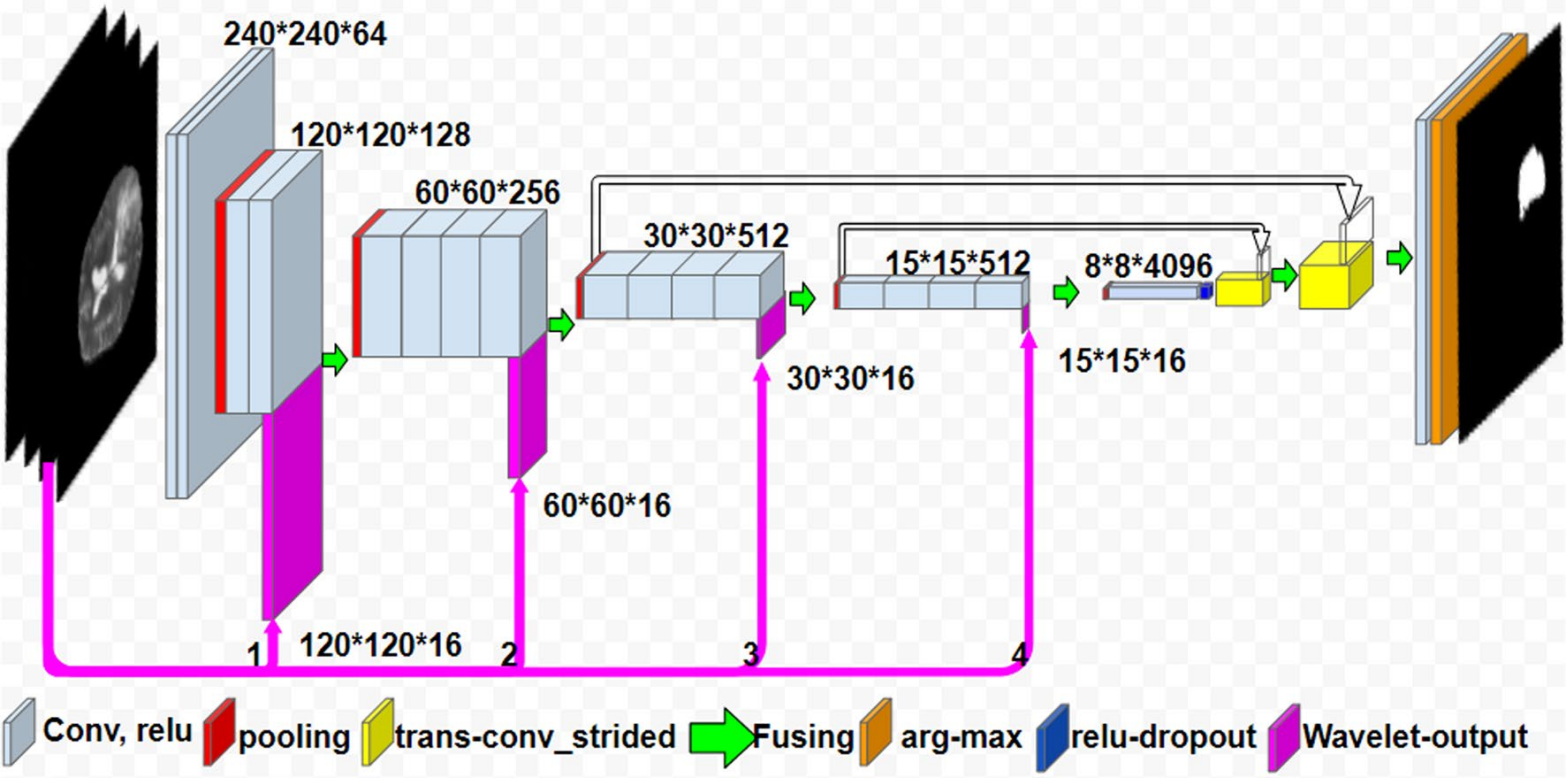
Four Wavelet injection path

A comparison of wavelet transforms output shows that db1 has a good performance in edges identification (similar to the derivative operator) also it has lower compression time compared to other transforms. In fact, it evokes a suitable balance between performance in edge detection and time-consuming. Due to the relative size of the tumor to the input image (250*250 pixel), the wavelet transforms outputs with sizes smaller than 15 × 15 actually did not contain signs of tumor, and no further compression was required. For this reason, sizes 120, 60, 30 and 15 were selected as target layer sizes for the injection of wavelet output. Therefore, four paths with wavelet injection capability were considered (Fig. 6) and their performance was evaluated as Table 2.

**Table.2:** Results of 4 Wavelet injection path

Architecture	Dice evaluation (%)
Base FCN architecture	77.9
WFCN1(1st level injection: path number 1 in the Figure)	91.8
WFCN2(2nd level injection: path number 2 in the Figure)	91.4
WFCN3(3rd level injection): path number 3 in the Figure	90.3
WFCN4(4th level injection): path number 4 in the Figure	90.4

WFCN1 architecture, which refers to the use of first-level wavelet injection (with 120*120 size) in the CNN structure, was chosen as the top model among other WFCNs. Evaluation with details of WFCN1 architecture are represented in Table 3:

**Table.3:** WFCN1 detailed performance

Evaluation	Value (%)
Dice	91.8
Dice variance	0.1
Pixel accuracy	99
Mean pixel accuracy	96
Sensitivity	93
Specificity	99
AUC	97

**Fig. 7:**
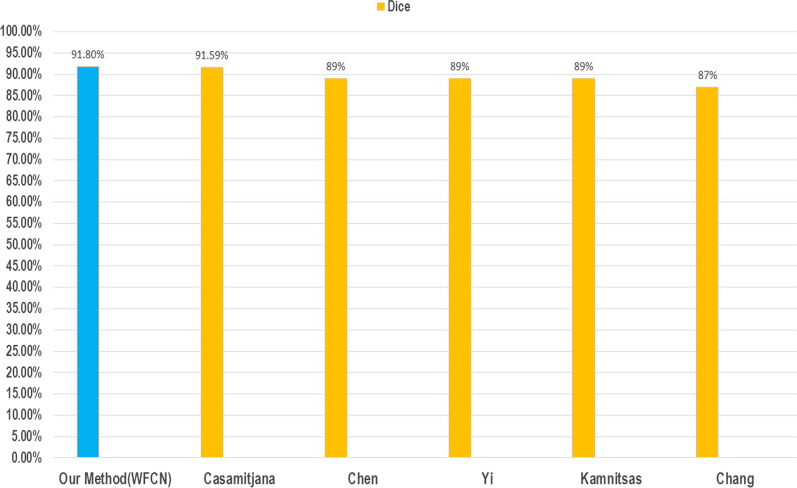
Comparison of the accuracy of the method presented in this study against other methods

A comparative surveying the related studies [55–59] show our methods achievement in the term of Dice accuracy. Figure 7 demonstrates the performance comparison between the superior ones from the surveyed studies (yellow columns) and our method (blue column).

## Discussion and conclusion

As mentioned earlier, various functions can be used as a wavelet kernel, and in fact, a wide variety of functions can be used in this regard. Db1 as a wavelet kernel is useful in identifying the edges of the brain tumor, so it is a good choice for brain tumor segmentation. Also, it achieves a good balance between the computational burden and edge detection. Other studies on the application of the combinational models of CNNs and wavelet transforms show that different functions have been employed as the wavelet kernels. Such as Haar [60], daubechies3 and symmlet4 [61], Gabor [62, 63], Contourlet [40], and Curvelet [64]. The main reason for such variation is based on the different nature of the applications and also the difference in the input data.

The first point about the wavelet kernel selected in this study is its simplicity. Means that less computation is required to use this function as the wavelet kernel, which speeds up the using of wavelet transforms on the images. Therefore applying of this function as a wavelet kernel does not add much time overload to the process of model training. The time overload associated with applying wavelet transforms must be considered in the term of time complexity. One of the solutions to reduce computation at this stage is to applying wavelet transforms in the first iteration of learning, saving the results and using them in the next iterations. However, it is important to pay attention to memory management in such situations. The second point is about the shape of the db1. Since db1’s shape is similar to the derivative operator and edge is a key feature to identify tumor areas from non-tumor backgrounds in brain scan image, the ability of a function to mimic the derivative operator and discover the edges of the image is considered a major advantage for db1.

The findings of the study, can help researcher’s in better designing of CNNs with the aim of brain tumor segmentation. Because the difference in brightness levels between tumors and healthy areas of the brain in the MRI images, db1 can be used as an auxiliary part of CNNs. Due to its low computational cost, it can be useful in designing rapid tumor segmentation methods. It can also be used on lower computing capacity devices and responds in real time.

In the end, it should be noted that some additional paths can improve CNN performance in a desired task and It is better not to compromise the core network structure during these additions. In fact, in such a situation we can expect that additional extension causes improved performance. Depending on the nature of the CNN, and its ability to select from the inputs, if the additional path is a performance enhancer, it has been remained and interfered within the model training process. Otherwise, it will be removed from the training process. Consequently, by maintaining the basic structure of the CNN model, considering the appropriate wavelet function, management of computation time and the number of decomposition levels, the network performance can be improved.

### Data availability statement

The **Bra**in **T**umor **S**egmentation dataset (BRATS) provided by NIH is used for testing the research idea: around 155 slices for each patient in.mha format as a collection of images: T1, T2, T1 with contrast (T1C), Flair and a Ground Truth segmented image. BRATS 2015 used in this study contains 224 High Grade glioma and 50 Low Grade Glioma MRI images.

### Limitations

The computing power required to process deep learning models is high, and researchers were limited in their access to hardware and only had access to an average one (GPU: GTX 980Ti). This limited the study in terms of time and the ability to use heavier models, and researchers had to consider this issue in designing and testing the model.

Of course, today, with the advancement of technology in this field, the cost of access to more powerful hardware is gradually decreasing, and on the other hand, services are provided that can potentially be used in the design and implementation of similar studies by researchers.

## Data Availability

We used BRATS2015 dataset fro training and testing models. Brats2015 can be downloaded from: https://www.smir.ch/BRATS/Start2015#!#download. Accessing to the dataset using following login information: User: alizade.behruz@gmail.com. Pass: 123@AbC#321. Also granted permissions can be found at http://www.braintumorsegmentation.org/ documentations.

## References

[1] Jazayeri SB, Rahimi-Movaghar V, Shokraneh F, Saadat S, Ramezani R (2013). Epidemiology of primary CNS tumors in Iran: a systematic review. Asian Pac J Cancer Prev.

[2] Bashiri A, Ghazisaeedi M, Safdari R, Shahmoradi L, Ehtesham H (2017). Improving the prediction of survival in cancer patients by using machine learning techniques: experience of gene expression data: a narrative review. Iran J Public Health.

[3] Logeswari T, Karnan M. An improved implementation of brain tumor detection using soft computing. In: 2010 Second international conference on communication software and networks: 2010: IEEE; 2010. p. 147–151.

[4] Maiti I, Chakraborty M. A new method for brain tumor segmentation based on watershed and edge detection algorithms in HSV colour model. In: 2012 National conference on computing and communication systems: 2012: IEEE; 2012. p. 1–5.

[5] Varsha Y, Shyry SP (2014). A novel approach for identifying the stages of brain tumor. Int J Comput Trends Technol IJCTT.

[6] Yang W, Siliang M. Automatic detection and segmentation of brain tumor using fuzzy classification and deformable models. In: 2011 4th International conference on biomedical engineering and informatics (BMEI): 2011: IEEE; 2011. p. 1680–1683.

[7] Heiss W-D, Raab P, Lanfermann H (2011). Multimodality assessment of brain tumors and tumor recurrence. J Nucl Med.

[8] Moon N, Bullitt E, Van Leemput K, Gerig G. Automatic brain and tumor segmentation. In: International conference on medical image computing and computer-assisted intervention: 2002: Springer; 2002. p. 372–379.

[9] Guoqiang W, Dongxue W. Segmentation of brain MRI image with GVF snake model. In: 2010 First international conference on pervasive computing, signal processing and applications: 2010: IEEE; 2010. p. 711–714.

[10] Bara S, El Maia H, Hammouch A, Aboutajdine D. A robust approach for the detection of brain tumors by variational b-spline level-set method and brain extraction. In: 2014 International conference on multimedia computing and systems (ICMCS): 2014: IEEE; 2014. p. 62–68.

[11] Savareh BA, Emami H, Hajiabadi M, Azimi SM, Ghafoori M (2019). Wavelet-enhanced convolutional neural network: a new idea in a deep learning paradigm. Biomed Eng Biomed Tech.

[12] Bashiri A, Savareh BA, Ghazisaeedi M (2019). Promotion of prehospital emergency care through clinical decision support systems: opportunities and challenges. Clin Exp Emerg Med.

[13] Toga AW, Thompson PM, Mega MS, Narr KL, Blanton RE (2001). Probabilistic approaches for atlasing normal and disease-specific brain variability. Anat Embryol.

[14] Prastawa M, Bullitt E, Ho S, Gerig G (2004). A brain tumor segmentation framework based on outlier detection. Med Image Anal.

[15] El-Melegy MT, Mokhtar HM (2014). Tumor segmentation in brain MRI using a fuzzy approach with class center priors. EURASIP J Image Video Process.

[16] Amsaveni V, Singh NA. Detection of brain tumor using neural network. In: 2013 Fourth international conference on computing, communications and networking technologies (ICCCNT): 2013: IEEE; 2013. p. 1–5.

[17] Schmidt M, Levner I, Greiner R, Murtha A, Bistritz A. Segmenting brain tumors using alignment-based features. In: Fourth international conference on machine learning and applications (ICMLA'05): 2005: IEEE; 2005. p. 6.

[18] Sachin GN, Khairnar V. Brain tumor detection based on symmetry information. arXiv e-prints: arXiv:1401.6127 2013.

[19] Constantin AA, Bajcsy BR, Nelson CS. Unsupervised segmentation of brain tissue in multivariate MRI. In: 2010 IEEE international symposium on biomedical imaging: from nano to macro: 2010: IEEE; 2010. p. 89–92.

[20] Kobashi S, Matsui M, Inoue N, Kondo K, Sawada T, Hata Y. Adaptive brain tissue classification with fuzzy spatial modeling in 3T IR-FSPGR MR images. In: 2006 World automation congress: 2006: IEEE; 2006. p. 1–6.

[21] Yu C-P, Ruppert G, Collins R, Nguyen D, Falcao A, Liu Y. 3D blob based brain tumor detection and segmentation in MR images. In: 2014 IEEE 11th international symposium on biomedical imaging (ISBI): 2014: IEEE; 2014. p. 1192–1197.

[22] Karnan M, Logheshwari T. Improved implementation of brain MRI image segmentation using ant colony system. In: 2010 IEEE international conference on computational intelligence and computing research: 2010: IEEE; 2010. p. 1–4.

[23] Idrissi N, Ajmi FE. A hybrid segmentation approach for brain tumor extraction and detection. In: 2014 International conference on multimedia computing and systems (ICMCS): 2014: IEEE; 2014. p. 235–240.

[24] Charutha S, Jayashree M. An efficient brain tumor detection by integrating modified texture based region growing and cellular automata edge detection. In: 2014 International conference on control, instrumentation, communication and computational technologies (ICCICCT): 2014: IEEE; 2014. p. 1193–1199.

[25] Fazli S, Nadirkhanlou P. A novel method for automatic segmentation of brain tumors in MRI images. arXiv preprint 13127573 2013.

[26] Kumar M, Mehta KK: A texture based tumor detection and automatic segmentation using seeded region growing method. Int J Comput Technol Appl 2011;2(4).

[27] Özyurt F, Sert E, Avci E, Dogantekin E (2019). Brain tumor detection based on Convolutional Neural Network with neutrosophic expert maximum fuzzy sure entropy. Measurement.

[28] Özyurt F, Sert E, Avcı D (2020). An expert system for brain tumor detection: Fuzzy C-means with super resolution and convolutional neural network with extreme learning machine. Med Hypotheses.

[29] Selvathi D, Anitha J. Effective fuzzy clustering algorithm for abnormal MR brain image segmentation. In: 2009 IEEE international advance computing conference: 2009: IEEE; 2009. p. 609–614.

[30] Vijay J, Subhashini J. An efficient brain tumor detection methodology using K-means clustering algoriftnn. In: 2013 International conference on communication and signal processing: 2013: IEEE; 2013. p. 653–657.

[31] Havaei M, Davy A, Warde-Farley D, Biard A, Courville A, Bengio Y, Pal C, Jodoin P-M, Larochelle H (2017). Brain tumor segmentation with deep neural networks. Med Image Anal.

[32] Lyksborg M, Puonti O, Agn M, Larsen R. An ensemble of 2D convolutional neural networks for tumor segmentation. In: Scandinavian conference on image analysis: 2015: Springer; 2015. p. 201–211.

[33] Havaei M, Dutil F, Pal C, Larochelle H, Jodoin P-M. A convolutional neural network approach to brain tumor segmentation. In: BrainLes 2015: 2015: Springer; 2015. p. 195–208.

[34] Zhao L, Jia K. Deep feature learning with discrimination mechanism for brain tumor segmentation and diagnosis. In: 2015 international conference on intelligent information hiding and multimedia signal processing (IIH-MSP): 2015: IEEE; 2015. p. 306–309.

[35] Kamnitsas K, Ledig C, Newcombe VF, Simpson JP, Kane AD, Menon DK, Rueckert D, Glocker B (2017). Efficient multi-scale 3D CNN with fully connected CRF for accurate brain lesion segmentation. Med Image Anal.

[36] Dvořák P, Menze B. Local structure prediction with convolutional neural networks for multimodal brain tumor segmentation. In: International MICCAI workshop on medical computer vision: 2015: Springer; 2015. p. 59–71.

[37] Bengio Y (2009). Learning deep architectures for AI.

[38] Ciresan D, Giusti A, Gambardella L, Schmidhuber J (2012). Deep neural networks segment neuronal membranes in electron microscopy images. Adv Neural Inf Process Syst.

[39] Pan W, Bui TD, Suen CY. Isolated handwritten Farsi numerals recognition using sparse and over-complete representations. In: 2009 10th international conference on document analysis and recognition: 2009: IEEE; 2009. p. 586–590.

[40] Kang J, Lu C, Cai M, Zhang W-Q, Liu J. Neuron sparseness versus connection sparseness in deep neural network for large vocabulary speech recognition. In: 2015 IEEE international conference on acoustics, speech and signal processing (ICASSP): 2015: IEEE; 2015. p. 4954–4958.

[41] Boussion N, Hatt M, Lamare F, Bizais Y, Turzo A, Cheze-Le Rest C, Visvikis D (2006). A multiresolution image based approach for correction of partial volume effects in emission tomography. Phys Med Biol.

[42] Sun J, Yao M, Xu B, Bel P (2011). Fabric wrinkle characterization and classification using modified wavelet coefficients and support-vector-machine classifiers. Text Res J.

[43] Srivastava R (2013). Research developments in computer vision and image processing: methodologies and applications: methodologies and applications.

[44] Savareh BA, Bashiri A, Behmanesh A, Meftahi GH, Hatef B (2018). Performance comparison of machine learning techniques in sleep scoring based on wavelet features and neighboring component analysis. PeerJ.

[45] Savareh BA, Ghanjal A, Bashiri A, Motaqi M, Hatef B (2017). The power features of Masseter muscle activity in tension-type and migraine without aura headache during open-close clench cycles. PeerJ.

[46] Menze BH, Jakab A, Bauer S, Kalpathy-Cramer J, Farahani K, Kirby J, Burren Y, Porz N, Slotboom J, Wiest R (2014). The multimodal brain tumor image segmentation benchmark (BRATS). IEEE Trans Med Imaging.

[47] Zhu W, Xiang X, Tran TD, Xie X. Adversarial deep structural networks for mammographic mass segmentation. arXiv preprint 161205970 2016.

[48] Graps A (1995). An introduction to wavelets. IEEE Comput Sci Eng.

[49] Vatansever F, Ozdemir A (2008). A new approach for measuring RMS value and phase angle of fundamental harmonic based on wavelet packet transform. Electr Power Syst Res.

[50] Douka E, Loutridis S, Trochidis A (2003). Crack identification in beams using wavelet analysis. Int J Solids Struct.

[51] Lee DT, Yamamoto A (1994). Wavelet analysis: theory and applications. Hewlett Packard J.

[52] Qian S, Yang Q. Graphical system and method for designing a mother wavelet. In*.*: Google Patents; 2000.

[53] Goswami JC, Chan AK (2011). Fundamentals of wavelets: theory, algorithms, and applications.

[54] Young RK (2012). Wavelet theory and its applications.

[55] Casamitjana A, Puch S, Aduriz A, Sayrol E, Vilaplana V. 3d convolutional networks for brain tumor segmentation. In: Proceedings of the MICCAI challenge on multimodal brain tumor image segmentation (BRATS) 2016. p. 65–68.

[56] Chen H, Dou Q, Yu L, Heng P-A. VoxResNet: Deep Voxelwise Residual Networks for Volumetric Brain Segmentation. arXiv preprint 160805895 2016. 10.1016/j.neuroimage.2017.04.041 28445774

[57] Yi D, Zhou M, Chen Z, Gevaert O. 3-D Convolutional Neural Networks for Glioblastoma Segmentation. arXiv preprint 161104534 2016.

[58] Chang PD. Fully convolutional deep residual neural networks for brain tumor segmentation. In: Brainlesion: glioma, multiple sclerosis, stroke and traumatic brain injuries: second international workshop, BrainLes 2016, with the challenges on BRATS, ISLES and mTOP 2016, held in conjunction with MICCAI 2016, Athens, Greece, October 17, 2016, revised selected papers. Edited by Crimi A, Menze B, Maier O, Reyes M, Winzeck S, Handels H. Cham: Springer International Publishing; 2016. p. 108–118.

[59] Kamnitsas K, Ferrante E, Parisot S, Ledig C, Nori A, Criminisi A, Rueckert D, Glocker B. DeepMedic on brain tumor segmentation. In: Athens, Greece Proc BRATS-MICCAI 2016.

[60] Fujieda S, Takayama K, Hachisuka T. Wavelet convolutional neural networks for texture classification. arXiv preprint 170707394 2017.

[61] Liu Y, Cheng J. Protein secondary structure prediction based on wavelets and 2D convolutional neural network. In: Proceedings of the 7th international conference on computational systems-biology and bioinformatics: 2016; 2016. p. 53–57.

[62] Kwolek B. Face detection using convolutional neural networks and Gabor filters. In: International conference on artificial neural networks: 2005: Springer; 2005. p. 551–556.

[63] Kleć M, Korzinek D (2015). Pre-trained deep neural network using sparse autoencoders and scattering wavelet transform for musical genre recognition. Comput Sci.

[64] Jadoon MM, Zhang Q, Haq IU, Butt S, Jadoon A (2017). Three-class mammogram classification based on descriptive CNN features. BioMed Res Int.

